# Fast growing angiosarcoma of the right atrium after radiofrequency catheter ablation: a missed diagnosis or misdiagnosis case report

**DOI:** 10.1186/s12885-019-6450-2

**Published:** 2020-01-06

**Authors:** Yi Yu, Qunshan Wang, Jian Sun, Jing Zhao, Suyun Chen, Yigang Li

**Affiliations:** 1 Department of Cardiology, Xinhua Hospital Affiliated with the School of Medicine, 1665 Kongjiang Road, Shanghai, 200092 People’s Republic of China; 2 Department of Pathology, Changhai Hospital Affiliated with the Second Military Medical University, Shanghai, China; 3 Department of Nuclear Medicine, Xinhua Hospital affiliated with the School of Medicine, Shanghai Jiaotong University, Shanghai, China

**Keywords:** Cardiac tumor, Cardiac imaging

## Abstract

**Background:**

Primary angiosarcomas of the right atrium are extremely rare, often resulted in missed diagnosis or misdiagnosis with routine examination tools. These malignant cardiac tumors are highly aggressive with generally poor prognosis. Surgical excision is the mainstay of treatment as it is essentially not responsive to current regimens of chemoradiotherapy.

**Case presentation:**

Herein, we describe a patient who initially presented with paroxysmal atrial fibrillation and was subsequently treated with radiofrequency catheter ablation (RFCA). Prior to RFCA, an initial transesophageal echocardiography revealed a local thickening of the intratrial septum. Three months later, she was hospitalized with progressive dyspnea and massive pericardial effusion. A large immobile, non-pedunculated mass, occupying almost half of the right atrium was detected by transthoracic and transesophageal echocardiogram. Multimodality cardiac imaging was useful in further characterizing this mass, which was ultimately diagnosed as an angiosarcoma based upon biopsy results. The growth rate was extremely rapid following RFCA, and patient underwent surgical excision. After discharge, the angiosarcoma recurred and patient survived for 7 months from the first episode of tamponade.

**Conclusions:**

Primary cardiac angiosarcoma of the right atrium can easily be mistaken for structural anomalies in its early stages, losing the opportunity for initiating earlier treatments to improve potential patient outcomes. The correct diagnosis of this rare case relied on the comprehensive utilization of multimodal imaging techniques including biopsy.

## Background

Angiosarcomas are malignant tumors that originate from the vascular endothelium. Incidence of angiosarcoma is extremely low [1], but angiosarcomas belong to the most frequently occurring and rapidly spreading primary cardiac tumor [2] Right heart is the usual location of the involvement of the disease, mostly in the right atrium, which is opposed to benign tumors which are usually left sided tumors. The majority of patients exhibit evidence of pericardial disease and/or cardiac failure [3]. Pericardial effusions may occur with tamponade, but are rarely seen at the initial presentation [4]. Despite treatment, the prognosis is usually dismal with a mean survival of 9–12 months after diagnosis [5].

## Case presentation

**Fig. 1:**
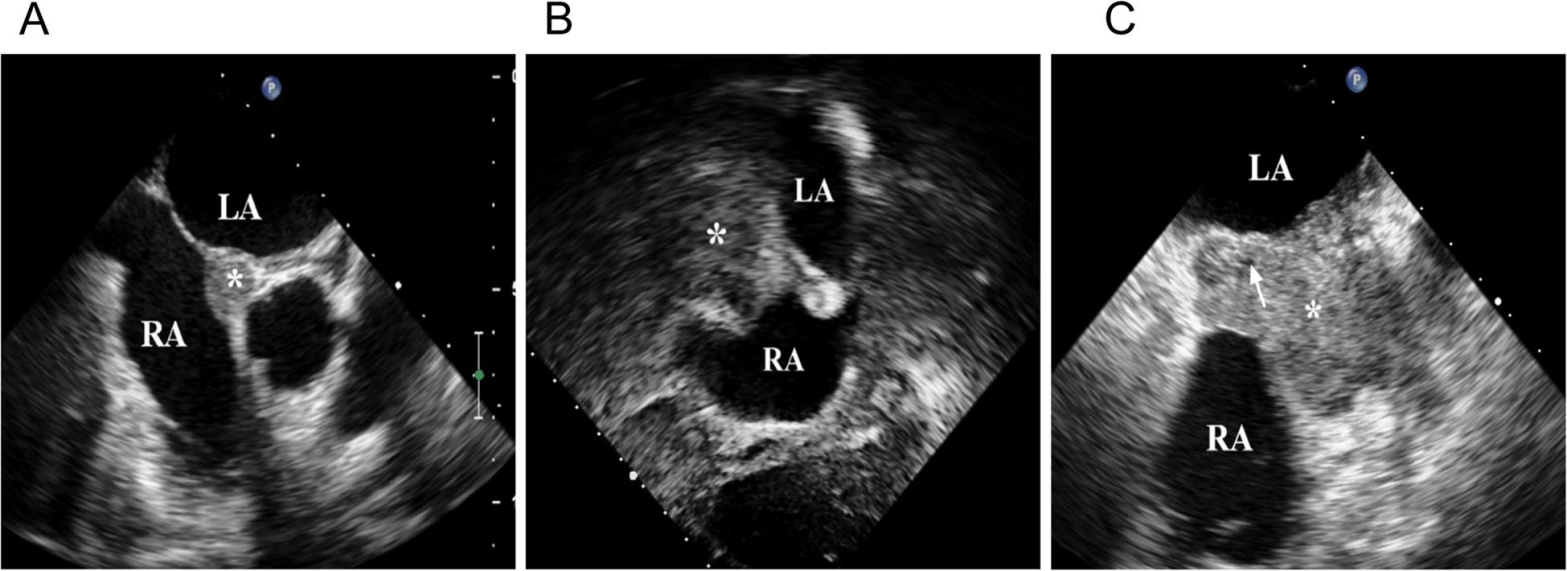
Echocardiography revealed the changes of the mass before and after RFCA. **a** Before RFCA, transesophageal echocardiography (TEE) revealed a local thickening of the interatrial septum (*) near the root of aorta. **b** Transthoracic echocardiography (TTE) detected a large immobile, non-pedunculated mass (*) involving the interatrial septum 3 months after RFCA. **c** TEE revealed a large polymorphic tumor (*) infiltrating the right atrial walls with hypoechoic area (arrow) 3 months after RFCA

A 60-year-old female is described who presented with atrial premature beats and paroxysmal atrial fibrillation. A transesophageal echocardiogram (TEE) was performed which showed a small, localized thickening of the interatrial septum that was considered as a structural variant (Fig. 1a). She subsequently underwent circumferential pulmonary vein radiofrequency ablation (RFCA)**.** Three months after RFCA, the patient was readmitted following the sudden onset of chest tightness, dyspnea, transient palpitation, and recurrent pericardial effusion. Chest x-ray examination revealed moderate cardiomegaly and T-wave abnormalities in precordial leads were found in electrocardiogram. Transthoracic echocardiography (TTE) examination revealed a large immobile, non-pedunculated mass (47 mm × 17 mm × 11 mm) occupying almost half of the right atrium (RA) (Fig. 1b) and extending along the lateral wall of the RA to the interatrial septum. The mass did not extend into the superior vena cava (SVC) and did not cause stenosis of the tricuspid valve annulus. Transesophageal echocardiogram (TEE) confirmed the presence of the mass and showed that it extended from the orifice of SVC to the tricuspid valve annulus and from the right auricle (RAA) to the RA (Fig. 1c). The mass exhibited central cavitation suggesting that blood flow was poor in this region. No malignant or atypical cells were found in the bloody pericardial effusion.

**Fig. 2:**
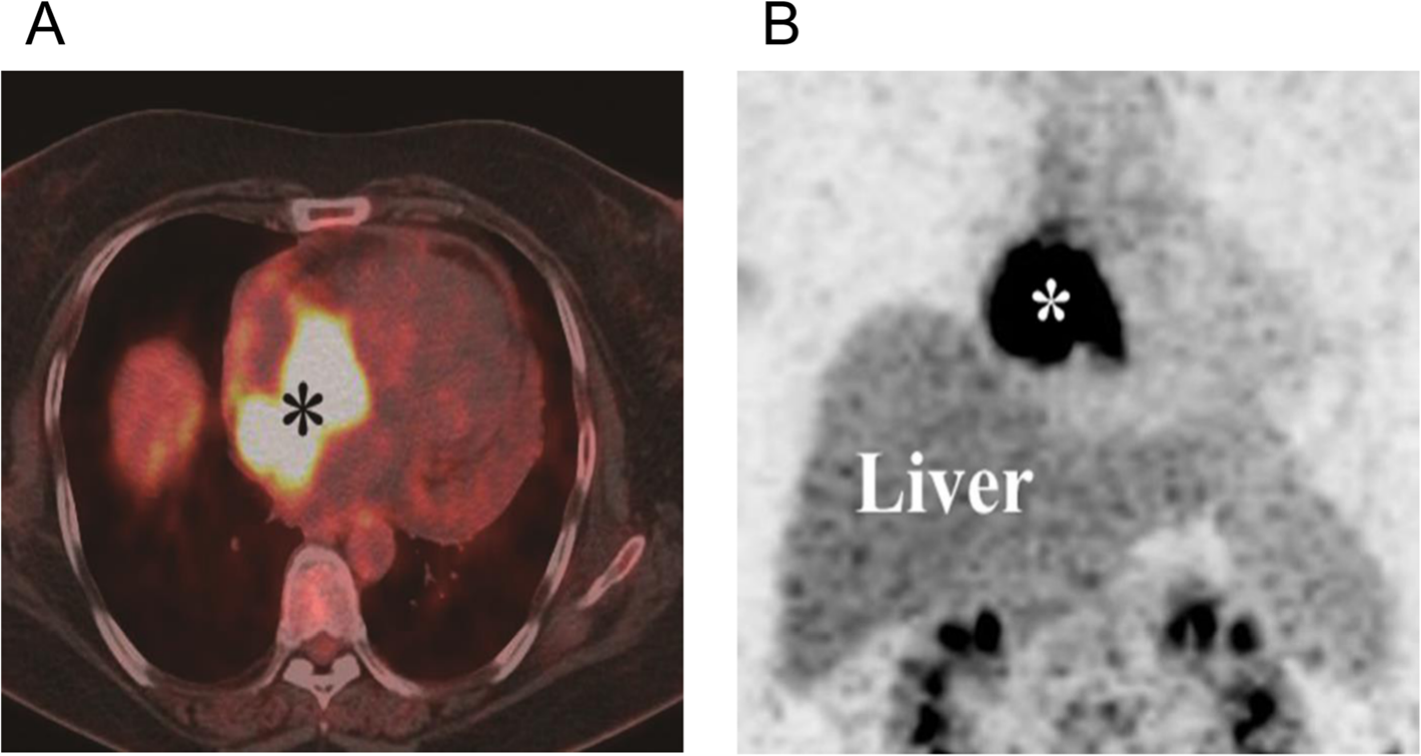
PET-CT assessed the metabolic activity of the cardiac mass. **a** Positron emission tomography scan showed hypermetabolic activity (*) in the RA with a standard uptake value SUV_max_ of 21.2. **b** 18F-FDG PET-CT fusion image illustrates high FDG uptake of the cardiac mass (*). 18F-FDG PET-CT: F18-fluorodeoxy glucose-positron emission tomography

**Fig. 3:**
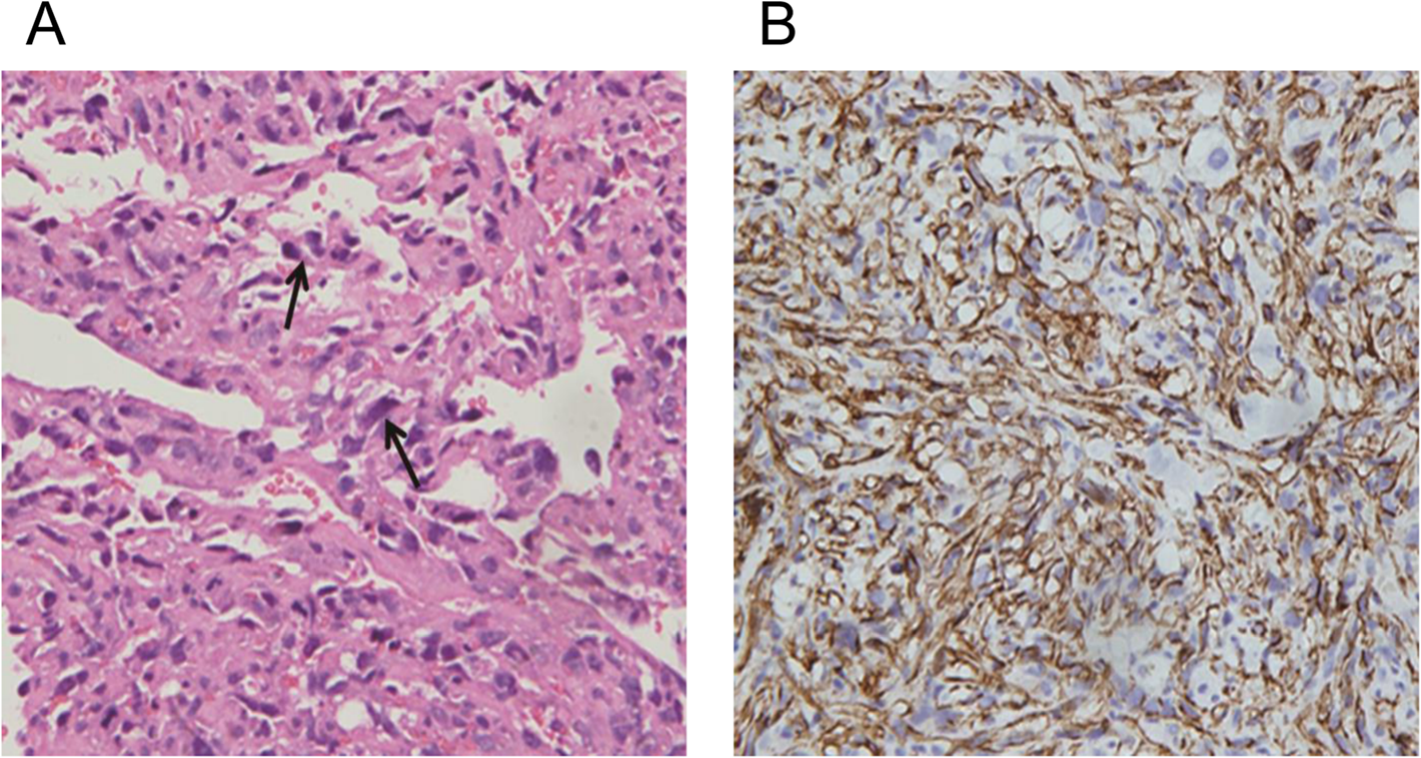
Pathological analysis identified the tumor as a primary cardiac angiosarcoma. **a** Malignant cells are frankly atypical with hyperchromatic nuclei (arrow). They line poorly formed immature vessels containing red blood cells (H&E stain). **b** Immunohistochemical stain for CD31 (blue) revealed the endothelial nature of the tumor cells (original magnification × 200)

Based upon these findings, our attention focused on the identity of the mass: cardiac tumor or hematoma? If it was a tumor, was it benign or malignant? If it was malignant, had it already metastasized? A F18-fluorodeoxy glucose-positron emission tomography (18F-FDG PET-CT) scan was advised to further characterization of the mass and assessment of metabolic activity. The scan revealed an area of abnormal tracer activity in a hard tissue mass occupying the RA and encroaching on the adjacent left atrial roof (Fig. 2a, b). With contour and heterogeneous contrast enhancement, the infiltrative mass was observed to arise from the RA. Moreover, another area of increased radiotracer activity was found in the RAA but there were no hypermetabolic lesions in adjacent organs. These images suggested the possibility of an angiosarcoma without metastasis. Subsequently, the patient underwent surgical excision of the tumor. Pathological analysis identified the tumor was a primary cardiac angiosarcoma. Microscopic findings showed that the tumor was composed of frequent slit-like vascular channels and hyperchromatic, elongated cells with obvious nuclear atypia (Fig. 3a). The results of immunohistochemical investigation revealed that tumor cells stained positive for CD31 (Fig. 3b), CD34, VI antigen, and vimentin which confirmed the endothelial origin of the tumor. Unfortunately, the patient refused treatment with adjuvant radiotherapy, chemotherapy or immunotherapy. Within 3 months post surgery, the tumor recurred in the same location. The patient survived for a total of 7 months from the first episode of tamponade.

## Discussion and conclusions

Prevalence of primary angiosarcomas of the right atrium is extremely rare [1]. These malignant cardiac tumors are highly aggressive with generally poor prognosis. Arrhythmia is often one of the first clinical symptoms of angiosarcoma [6], which may occur secondary to local invasion by the tumor. Our patient initially presented with atrial premature beats and paroxysmal atrial fibrillation. Although a TEE showed a small, localized thickening of the interatrial septum, it was not considered to be relevant and her arrhythmia was treated by RFCA.

Three months later at a follow-up visit a fully developed angiosarcoma complicated by right atrial wall invasion and pericardial tamponade were found. No malignant or atypical cells were found in the bloody pericardial effusion. Analysis of pericardial fluid does not always show malignant cells even in the presence of pericardial invasion which emphasizes the limited diagnostic value of this test [7]. Reviewing the previous TEE, a small local lesion may have existed at initial presentation, but considered as a local thickening of the interatrial septum at that time. A small primary angiosarcoma is therefore likely to yield a missed diagnosis or misdiagnosis.

There has been increased use of noninvasive imaging techniques for diagnosing the presence of tumors [8]. While echocardiography is usually the first line technique for imaging cardiac tumors, computed tomography (CT), MRI and PET-CT are more useful for detailed characterization and differential diagnosis. Echocardiographic imaging of the atrial septum can be challenging in adult patients. Specifically, **s**mall local lesions of the atrial septum are generally difficult to detect by TTE, because of low spatial resolution and poor penetration through the chest wall. TEE is a useful imaging modality with high resolution as the proximity between the transducer and heart, which could provide a superior evaluation of the tissue characteristics compared to TTE [9]. MRI is also a useful method which generally provides more diagnostic information regarding cardiac masses than PET-CT but there was no MRI instrument specializing in cardiac examination in our hospital at the time of initial presentation.

Due to its more restricted availability and higher cost, 18F-FDG PET-CT has been reserved as a non-invasive imaging modality for suspected metastasis of unknown origin and for preoperative staging of various neoplasms. It offers a high sensitivity scan for metabolic activity with precise anatomical localization with a better analysis of soft tissues. It enables assessment of the tumor’s infiltration of the myocardial walls, the pericardium and adjacent organs. This is valuable since malignant neoplasms and their metastases are always characterized by enhanced glucose utilization and therefore are detectable by 18F-FDG PET-CT.

Complete surgical excision provides the greatest survival benefit and outcome of the patient depends on if there is a recurrence of the tumor or not [10]. It is to note that surgery is not always an effective treatment as complete removal of the tumor could not be achieved in some patients due to tumor infiltration into adjacent cardiac tissue [10]. Moreover, the tumors are not encapsulated and can be very difficult to resect completely due to their poorly defined borders. Many surgeons consider such patients as having a poor long-term prognosis, but surgery is necessary to relieve the recurrent pericardial effusions and severe symptoms of cardiac tamponade produced by the tumor. Our patient survived only 7 months from the first episode of tamponade. An advanced cardiac angiosarcoma without metastases developed in our patient over a 3-month period after RFCA. To our knowledge, there have been no previous reports of cases with the rapid growth of a cardiac angiosarcoma after RFCA.

A large angiosacroma without metastasis, the diagnosis was suggested by 18F-FDG PET-CT, is highly unusual as these tumors are generally very aggressive and highly invasive [11] Sabzi et al. [12] reported an association of right atrial angiosarcoma with pericardial effusion and low cardiac ejection fraction, these phenomenon were not observed in our patient. On this basis of this discussion, we hypothesize that RFCA in conjunction with transseptal puncture to the tumor might contribute to the very rapid tumor growth in this patient.

In conclusion, primary cardiac angiosarcoma of the right atrium is an extremely rare but a highly aggressive tumor. In its early stages, these tumors can easily be mistaken for structural anomalies. The successful diagnosis of our case relied on the comprehensive utilization of multiple imaging techniques including biopsy examinations.

## Data Availability

All data generated or analysed during this study are included in this published article.

## Abbreviations

18F-FDG PET-CT: F18-fluorodeoxy glucose-positron emission tomography
CT: Computed tomography
RA: Right atrium
RAA: Right auricle
RFCA: Radiofrequency catheter ablation
SVC: Superior vena cava
TEE: Transesophageal echocardiogram
TTE: Transthoracic echocardiogram
